# Biological control of *Botrytis cinerea* in tomato and *Fusarium graminearum* in wheat using the novel species *Burkholderia mycopellens* and *Burkholderia crassaminum*

**DOI:** 10.3389/fmicb.2026.1776517

**Published:** 2026-04-10

**Authors:** Ayesha Kousar, Eliza Depoorter, Charlotte Peeters, Kurt Houf, Kris Audenaert, Peter Vandamme, Noémie De Zutter

**Affiliations:** 1 Laboratory of Microbiology, Department of Biochemistry and Microbiology, Faculty of Sciences, Ghent University, Ghent, Belgium; 2 Laboratory of Applied Mycology and Phenomics, Department of Plants and Crops, Faculty of Bioscience Engineering, Ghent University, Ghent, Belgium; 3 Department of Veterinary and Biosciences, Faculty of Veterinary Medicine, Ghent University, Merelbeke, Belgium

**Keywords:** biocontrol, *Botrytis cinerea*, *Burkholderia* spp., *Fusarium graminearum*, genomics, phenomics

## Abstract

The use of beneficial microbes to reduce plant stress upon fungal pathogens is a promising strategy for sustainable crop protection. In this study, we evaluated five novel *Burkholderia* strains for their capacity to mitigate *Botrytis cinerea* infection in tomato (*Solanum lycopersicum* L.) and *Fusarium graminearum* infection in wheat (*Triticum aestivum* L.). All strains significantly suppressed *B. cinerea* infection symptoms in tomato plants, hallmarked by a smaller decrease in photosynthetic activity and chlorophyll content compared to infected control. Using a GFP-tagged *F. graminearum* strain, we show that the strains reduced fungal biomass accumulation in *F. graminearum*-infected wheat leaves and mitigated chlorosis. Phytotoxicity assessments revealed no adverse effects in tomato for any strain, while two strains induced a mild reduction in chlorophyll fluorescence in wheat, suggesting potential host-specific phytotoxicity. Whole-genome sequencing of the *Burkholderia* strains revealed a rich repertoire of biosynthetic gene clusters (BGCs) with conserved replicon positioning. While all strains shared BGCs for known bioactive metabolites such as occidiofungin A, ornibactin, and pyochelin, variation in other clusters did not directly correlate with the phenotypic effects observed. Our results highlight the strong biocontrol potential *Burkholderia* strains in two economically important crops against two globally important fungal pathounder controlled conditions. Finally, a microbial phylogenomic analysis revealed that the five strains belong to two new and previously uncharacterized species within the *Burkholderia cepacia* complex, for which the names *Burkholderia mycopellens* sp. nov. and *Burkholderia crassaminum* sp. nov. are proposed. These strains hold promise as next-generation biocontrol agents for enhancing crop health and managing fungal diseases sustainably.

## 1 Introduction

*Burkholderia* bacteria occur naturally in soil, water, plants and the plants’ rhizosphere. They have great potential for agricultural applications due to their plant growth-promoting and biopesticidal properties (Pal et al., 2022; Xue et al., 2022). The ability of *Burkholderia* bacteria to synthesize a wide range of metabolites is rooted in their large, multi-chromosome genomes which typically consist of a primary chromosome and one or more secondary replicons or megaplasmids (Kunakom and Eustáquio, 2019). This multi-replicon nature of *Burkholderia* genomes plays an important role in their capacity to produce bioactive compounds as each replicon can harbor genes for the biosynthesis of secondary metabolites (Mannaa et al., 2019). Thus far, more than 80 different antimicrobial compounds have been reported from *Burkholderia* strains (Kunakom and Eustáquio, 2019; Rodríguez-Cisneros et al., 2023), some of which are strain specific (Quan et al., 2006), while others, including phenazines and ornibactin, are widely distributed (Rodríguez-Cisneros et al., 2023).

Within the genus *Burkholderia,* the *Burkholderia cepacia* complex (Bcc) comprises at least 27 closely related but genetically distinct species and many strains with an undefined species status (Vandamme and Peeters, 2014; Schoch et al., 2020; Esho and Othman, 2024). The biotechnological potential of several well-characterized *Burkholderia* and Bcc species has been extensively documented (Bevivino et al., 2000; Li et al., 2002; Chiarini et al., 2006; Depoorter et al., 2021b; Wang L. et al., 2023; Yang et al., 2024) and includes their use as biological agents to control phytopathogenic fungi, bacteria, protozoa and nematodes in tomato, wheat, corn, cotton, pea and grapevine among others (Cain et al., 2000; Perin et al., 2006). Despite their biotechnological potential, the use of *Burkholderia* species raises important biosafety concerns, particularly *B. pseudomallei* and those within the *B. cepacia* complex. Several species are recognized as opportunistic human and animal pathogens associated with respiratory and systemic infections in immunocompromised individuals (Blanchard and Waters, 2022; Wang et al., 2025). This dual beneficial-risk nature has led to regulatory restrictions in some countries and emphasizes the need for careful species- and strain-level characterization before agricultural application (Compant et al., 2008; Mendes et al., 2007; Sandanakirouchenane et al., 2017).

Despite the extensive work on several well-characterized Bcc members, a number of phylogenetically distinct groups within the complex remain taxonomically unresolved and functional under-characterized (Depoorter et al., 2020; Vandamme and Peeters, 2014). These poorly documented lineages lack integrated analyses combining comparative genomics, biosynthetic potential, and experimental validation of antifungal activity against major plant pathogens. This gap is particularly critical because strain-level diversity within the Bcc can strongly influence both metabolite production and biocontrol efficacy (Depoorter et al., 2016; Eberl and Vandamme, 2016).

In the present study, we analyzed the biocontrol potential of two poorly documented groups of Bcc strains with an undefined species status (Vandamme and Peeters, 2014). More specifically, we performed comparative genomic analyses coupled with *in vitro* and *in vivo* experiments to clarify their taxonomic status and investigate their biocontrol potential against two of the top 10 fungal pathogens in plant pathology (Dean et al., 2012). We examined their ability to control *Botrytis cinerea*, the causal agent of gray mold disease in more than 200 dicotyledonous plant species (Kilani-Feki and Jaoua, 2011), and *Fusarium graminearum*, the causal agent of *Fusarium* head blight in small grain cereal crops (Li et al., 2020). We hypothesized that these unclassified Bcc groups harbor distinct biosynthetic gene clusters (BGCs) linked with antifungal metabolite production, and that such genomic differences would correspond to measurable differences in biocontrol activity (Challis, 2008; Medema and Fischbach, 2015). Hence, we combined a comparative genomics approach with *in vitro* and *in vivo* assays. Genome mining enabled us to predict the biosynthetic capacity, while phenotyping experiments were essential to confirm whether the predicted pathways translated into observable antifungal effects.

## 2 Materials and methods

### 2.1 *Burkholderia* strains

Bacterial strains LMG 21824^T^ and R-15933 (Other Bcc A), and LMG 32019^T^, R-36351, and R-18634 (Other Bcc F) (Vandamme and Peeters, 2014; Supplementary Table S1; section 5.1 and 5.2) were grown from −80 °C glycerol stocks on tryptic soy agar (TSA, Oxoid) for 48 h at 28 °C. Single colonies were collected for *in vitro* and *in vivo* assays using a 10 μL inoculation loop and transferred and homogenized into 2 mL phosphate buffered saline (PBS, pH 7.2–7.4; Sigma-Aldrich). The optical density at 600 nm of these cell suspensions was adjusted to 2.0.

### 2.2 Fungal pathogens

*Botrytis cinerea* strain R16 (Faretra and Pollastro, 1991) and a GFP-transformed *Fusarium graminearum* strain PH-1 (Tan et al., 2020) were routinely cultivated for mycelial growth on Potato dextrose agar (PDA, Sigma-Aldrich) for 7 days at 25 °C in the dark. To induce sporulation, both strains were cultivated on PDA for 10 days at 22 °C under a regime of 12 h dark and 12 h combined UVA-UVC light (2x TUV 8 W T5 and 1x TL 8 W BLB; Philips, Eindhoven, Netherlands). Conidia of *B. cinerea* R16 were harvested by adding dH_2_O + 0.01% Tween20 to the PDA plates. Spores and mycelium were scrubbed from the plates with a Drigalski spatula. Conidia were separated from the mycelium using sterile Miracloth and were set at a concentration of 2 × 10^6^ spores per milliliter using a Bürker counting chamber. The spore suspension was diluted in a 54 mM phosphate buffer (0.05 M glucose, 54 mM phosphate buffer, pH 5.2) to a final concentration of 1 × 10^6^ spores/mL (Audenaert et al., 2002). Conidia of *F. graminearum* PH-1 were harvested by adding 20 mL of phosphate buffered saline (pH 7.2–7.4) supplemented with 0.01% Tween-80 to the PDA plates. Spores and mycelium were scrubbed from the plates with a Drigalski spatula. The suspension was filtered using sterile Miracloth and diluted in sterile PBS to a final concentration of 1 × 10^6^ spores per milliliter.

### 2.3 *In vitro* antagonism

The *in vitro* antagonism of the five *Burkholderia* strains was evaluated against *B. cinerea* R16 and *F. graminearum* PH-1 using a confrontation assay under direct and indirect contact. Under direct contact, vegetative mycelium of the actively growing plant pathogens was transferred as a 5-mm diameter plug to tryptic soy agar (TSA, Oxoid), after which four times 10 μL bacterial suspensions (OD_600_ 2.0) were spotted in a square at 40 mm from the fungal inoculation spot. Control plates were inoculated with sterile phosphate buffered saline instead of bacterial suspensions. All treatments and controls were included in triplicate. Plates were incubated at 28 °C for 5 days in the dark, after which fungal diameters and inhibition zones were evaluated by cell F software (IX81 microscope, IX2-BSW software version 01.03, OLYMPUS) and compared to the control. For the indirect contact assay, plates were prepared containing 4 mL of PDA mixed with 2 mL of fungal spore suspension. The plates were air-dried, after which a sterile membrane with a pore size of 0.1 μm was placed on the agar surface. Ten μL of bacterial suspensions (OD_600_ 2.0) was spotted in the center of the membrane. Control plates were inoculated with sterile PBS instead of bacterial suspensions. The plates were subsequently incubated at 28 °C for 5 days in the dark. All treatments and controls were included in triplicate. Mycelial growth inhibition of *B. cinerea* R16 and *F. graminearum* PH-1 was evaluated from 3 days onwards by measuring the zone of inhibition around the bacterial suspension spots.

### 2.4 *In vivo* biocontrol potential

The *in vivo* biocontrol potential of the five *Burkholderia* strains was evaluated against *B. cinerea* R16 on tomato leaf discs and against *F. graminearum* PH-1 on detached wheat leaves. Tomato (*Solanum lycopersicum* L. var. Marmande) plants were grown in standard potting soil (Structural, Type 0, potting soil for vegetables) for 28 days at 21 °C. Leaf discs with a diameter of 20 mm were obtained using a cork borer and were placed on their adaxial side in 24-well plates containing 2 mL sterile water. Subsequently, leaf discs were co-inoculated with 5 μL *B. cinerea* R16 spore suspension (1 × 10^6^ spores mL^−1^) and 5 μL bacterial suspension (OD_600_ 2.0). An infected control was included by replacing the bacterial suspension with sterile PBS. A non-infected, untreated control was included, which was inoculated with 10 μL PBS. The potential phytotoxic effect of the bacterial strains on the tomato leaf discs was assessed by co-inoculating 5 μL bacterial suspension with 5 μL PBS. All treatments and controls were included in 4 biological replicates (4 independent leaf discs). Leaf discs were incubated in a growth chamber at 21 °C with a photoperiod of 16 h.

Although the primary infection site of *F. graminearum* is the wheat ear, the use of a detached leaf assay as a high-throughput screening technique to assess *Fusarium* spp. infection has been well established in literature (Browne and Cooke, 2004; Purahong et al., 2012; Perochon and Doohan, 2016; Rafiei et al., 2025). The detached leaf assay was performed as described by Ameye et al. (2015) and as adapted by Tan et al. (2020). Wheat leaflets were co-inoculated with 5 μL *F. graminearum* PH-1 spore suspension (1 × 10^6^ spores. mL^−1^) and 5 μL bacterial suspension. An infected control, co-inoculated with PBS, and an untreated control, solely inoculated with PBS, were included in the experiment. The potential phytotoxic effect of the bacterial strains on wheat leaflets was evaluated by co-inoculating the bacterial suspensions with PBS. All treatments and controls were included in triplicate (three independent leaves). Detached leaves were incubated in a growth chamber at 21 °C with a 16 h photoperiod.

Disease progression was monitored in the tomato leaf discs and detached wheat leaves through multispectral imaging (CropReporter, Phenovation, The Netherlands) after 0 h, 24 h, 48 h, and 72 h, as described by Tan et al. (2020). The raw images were analyzed using the DataAnalysis 5.8.0 software (Phenovation, The Netherlands). Leaves or leaf discs were separated from the background using a chlorophyll-mask (level 200), while regions of interest (ROIs) on which the multispectral proxies were calculated were automatically drawn based on a matrix grid of a 6-well (detached leaf) or 24-well (leaf disc) plate. Several plant physiological traits were visualized throughout the experiment based on specific absorption, reflection, and emission patterns (Supplementary Table S2). Plant health was monitored using the proxies chlorophyll fluorescence (Fv/Fm), a measure of photosynthetic efficiency, and chlorophyll index (ChlIdx), a measure of chlorosis (Gitelson et al., 2003; Baker, 2008). Actively growing *F. graminearum* PH-1 biomass was monitored by means of the GFP signal corrected for foliar autofluorescence (cGFP), as specified by the manufacturer (Phenovation, The Netherlands).

### 2.5 Whole genome sequencing, assembly and annotation

Sample preparation was performed by cell harvesting, DNA extraction using the Maxwell 16 tissue DNA purification kit (Promega, United States), and subsequent DNA-quantification using the QuantiFluor ONE dsDNA quantification system (Promega, United States), as described by Depoorter et al. (2021a). Paired-end 150 bp libraries were sequenced on either an Illumina HiSeq 4000 sequencer (LMG 32019^T^, R-15933, and R-36351) or an Illumina NovaSeq 6000 sequencer (LMG 21824^T^ and R-18634) at the Oxford Genomics Centre (Wellcome Centre for Human Genetics, Oxford, United Kingdom). Adaptor trimming was done using fastp v0.23.2 (Chen et al., 2018).

Long-read sequencing libraries were prepared using the Oxford Nanopore Technologies (ONT) Native Barcoding kit SQK-NBD114 and sequenced on an Oxford Nanopore Technologies MinION instrument using a FLO-MIN114 flow cell. Base calling was done with Dorado v0.7.0^1^ with the dna_r10.4.1_e8.2_400bps_sup@v5.0.0 model for both simplex and duplex base calling. Reads were filtered using Chopper v0.3.0 retaining reads with a minimum length of 1,000 bp and a minimum quality score of 10 (De Coster and Rademakers, 2023). Quality control parameters of long-read sequencing data were assessed with NanoComp (De Coster and Rademakers, 2023). A consensus genome was assembled using Trycycler 0.5.5 (Wick et al., 2021). Filtered reads were subsampled using “trycycler subsample” into 12 subsets for a target genome size of 8.5 Mbp. Four independent read subsets were assembled using either Flye v2.9.2-b1786 (Kolmogorov et al., 2019), Raven v1.8.3 (Vaser and Šikić, 2021), or Miniasm v0.3-r179 (Li, 2016) and Minipolish v0.1.3 (Wick and Holt, 2019). Contigs from these assemblies were clustered and read depths were calculated using “trycycler cluster” by aligning the original filtered reads to each clustered contig. Contigs from clusters containing at least five contigs produced by at least two assemblers were reconciled using “trycycler reconcile.” Contigs from each reconciled cluster were subjected to multiple sequence alignment using “trycycler msa.” Original filtered reads were assigned to each cluster using “trycycler partition” with default minimum aligned length and minimum read coverage parameters and a consensus sequence was generated using “trycycler consensus.” Resulting assemblies were polished using Medaka v1.11.3^2^ using the r1041_e82_400bps_sup_v5.0.0 model and were polished using Polypolish v0.6.0 (Wick and Holt, 2022) using trimmed Illumina reads.

The genomic DNA G + C content and genome size were calculated using QUAST v5.2.0 (Gurevich et al., 2013). Completeness and contamination of assemblies were checked by CheckM v1.2.2 with the lineage set to the family Burkholderiaceae (Parks et al., 2015). Annotation was performed using Prokka v1.14.5 (Seemann, 2014). PlasmidHunter v1.4 was used to identify plasmids (Tian et al., 2024).

### 2.6 Detection of biosynthetic gene clusters

For the detection of biosynthetic gene clusters (BGCs) responsible for the production of secondary metabolites, antiSMASH v7.1.0 was used with strictness relaxed and all extra features (Blin et al., 2023). BiG-SCAPE v1.1.9 was used on antiSMASH GenBank gene cluster files with options “include_singletons,” “mix,” and “mibig” (Navarro-Muñoz et al., 2020). Result files from BiG-SCAPE “Network_Annotations_Full” and “mix_clustering_c0.30” were processed using R version 4.3.0. in RStudio version 2024.09.0 + 375 (R Core Team, 2013). The resulting cluster network was further refined by comparing gene topologies from network clusters of the same class and by splitting or merging network clusters where necessary. Heatmaps were generated using the ComplexHeatmap package v2.18.0 (Gu, 2022).

### 2.7 Publicly available genomes

The Genome Database Taxonomy toolkit (GTDB-Tk) v2.4.0 was used to assign taxonomic classifications of LMG 21824^T^ and LMG 32019^T^ using the “classify_wf” workflow with the GTDB r220 database (Chaumeil et al., 2022). Publicly available whole-genome sequences of three additional strains belonging to Other Bcc groups A (*Burkholderia* sp. AU30280 and BCC0405) (Tatusova et al., 2014; Mullins et al., 2020) and F (*Burkholderia* sp. D-99) (Tatusova et al., 2014), Bcc type strains, and *Burkholderia gladioli* ATCC 10248^T^ were downloaded from the NCBI database (23/05/2024).

### 2.8 Phylogenomic analyses

The amino acid sequences of 107 essential single-copy core genes were extracted using bcgTree v1.2.0 (Ankenbrand and Keller, 2016). A partitioned maximum-likelihood analysis was performed to construct a phylogenomic tree where *B. gladioli* ATCC 10248^T^ was included as an outgroup. To visualize the phylogenetic tree and make annotations, the interactive tree of life (iTOL) was used (Letunic and Bork, 2021). Average nucleotide identity (ANI) values were calculated through OrthoANI v1.2 using usearch v11.0.667 (Lee et al., 2016; Riesco and Trujillo, 2024). The type (strain) genome server (TYGS) was used to calculate pairwise digital DNA–DNA hybridization (dDDH) values (formula d4) and their confidence intervals (Meier-Kolthoff and Göker, 2019).

### 2.9 Biochemical characterization and cellular fatty acid analysis

Sample preparation, including cell harvesting and preparation, separation, identification and biochemical characterization of fatty acid methyl esters, was performed as described by Vandamme et al. (1992), Henry et al. (2001), and Depoorter et al. (2020).

### 2.10 Statistical analyses

All graphs were created in RStudio (v4.3.0) with the function *ggplot* (package ggplot2) (R Core Team, 2013). Statistical analyses were performed using IBM SPSS Statistics v25.0 (Armonk, New York). For both *in vitro* and *in vivo* experiments, treatments and controls were compared using ANOVA followed by Tukey’s *post-hoc* test. To evaluate phytotoxicity of the bacterial isolates on wheat or tomato, treated leaves/leaf discs were compared to the untreated blank by means of an independent sample *t*-test. The assumptions of normality and homoscedasticity were assessed using diagnostic plots. When substantial heteroscedasticity was detected, Welch’s correction was applied, and Dunnett’s T3 test was used for post-hoc comparisons. All hypotheses were tested at a 5% significance level.

## 3 Results

### 3.1 *In vitro* antagonism

**Figure 1:**
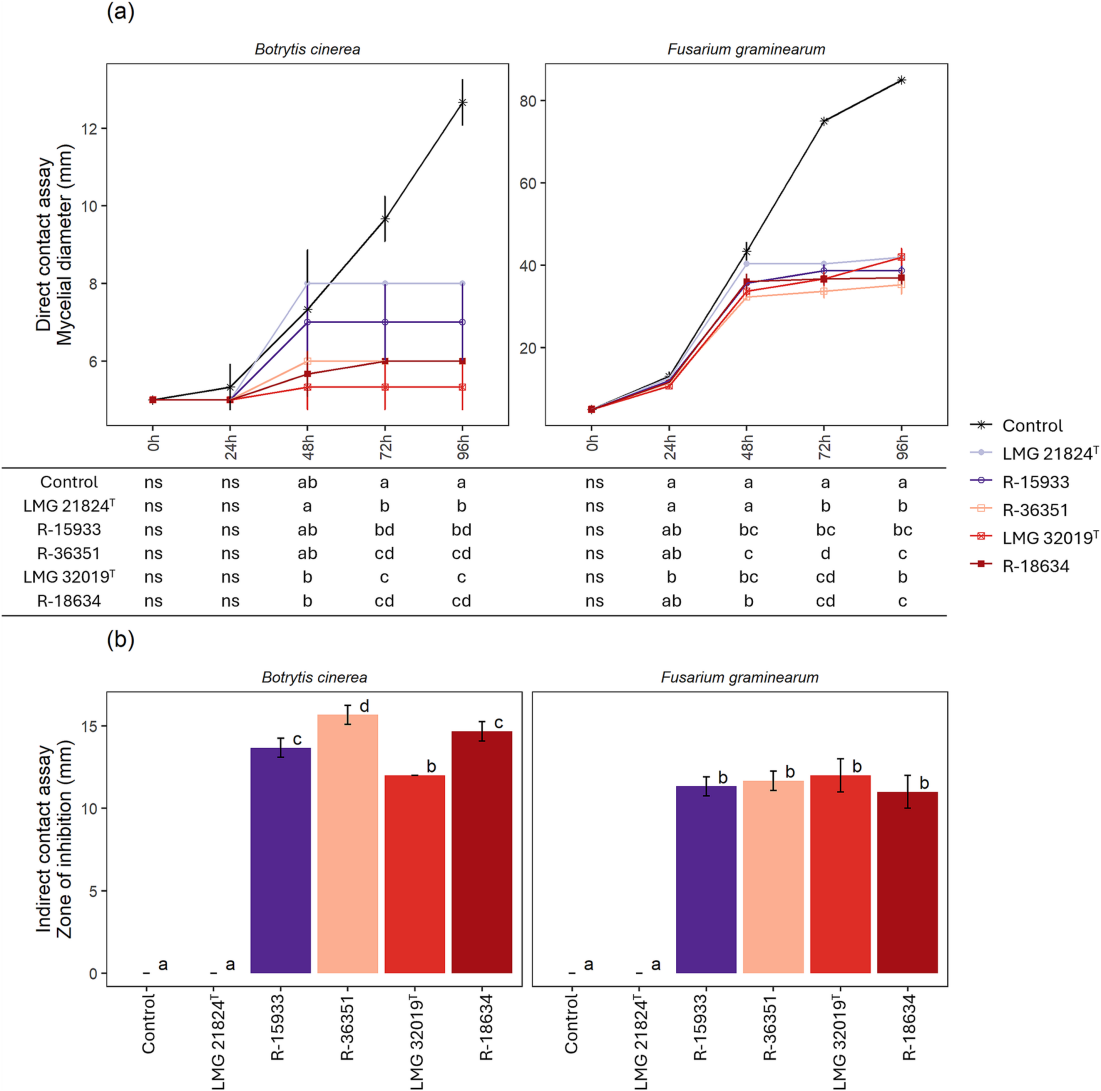
**(a)** *B. cinerea* R16 and *F. graminearum* PH-1 mycelial diameter (mm) in direct contact assay with *Burkholderia* strains. **(b)** Zone of inhibition (mm) against *B. cinerea* R16 and *F. graminearum* PH-1 under indirect contact with *Burkholderia* strains. Values represent mean ± SD of three biological replicates. Significant differences were calculated per timepoint as per ANOVA and post-hoc Tukey test and are indicated by means of significance letters. Different letters indicate significant differences at a 95%-confidence level.

In the direct contact assay, strains LMG 32019^T^ and R-18634 significantly inhibited *B. cinerea* R16 mycelial growth after 48 h, while after 72 h, all strains significantly inhibited *B. cinerea* R16 mycelial growth (Figure 1a; LMG 21824^T^, *p* = 0.022; R-15933, *p* = 0.017; R-36351, *p* = 0.011; LMG 32019^T^, *p* = <0.001; R-18634, *p* = 0.011). Strain LMG 32019ᵀ had the highest inhibitory effect on *B. cinerea* R16, limiting mycelial growth to 5 mm (control plates: 13 mm), while strain LMG 21824ᵀ had the lowest inhibitory effect, with mycelial growth limited to 8 mm. Under indirect contact, strains R-15933, R-36351, LMG 32019^T^ and R-18634 had a significant inhibitory effect on *B. cinerea* R16 mycelial growth, while strain LMG 21824^T^ showed no effect (Figure 1b; Supplementary Figure S1). In addition, strain LMG 32019^T^ significantly reduced mycelial growth of *F. graminearum* PH-1 after 24 h in the direct contact assay. After 48 h, strains R-36351, R-18634, and R-15933 significantly inhibited *F. graminearum* PH-1 mycelial growth, while strain LMG 21824^T^ significantly inhibited mycelial growth from 72 h onwards. After 96 h, the final mycelial diameters of *Fusarium graminearum* PH-1 were limited to 35 mm, 37 mm and 39 mm by strains R-36351, R-18634 resp. R-15933, and to 42 mm by strains LMG 21824^T^ and LMG 32019^T^ (control plates: 85 mm) (Figure 1a; Supplementary Figure S1). Under indirect contact between *F. graminearum* PH-1 and the bacteria, strains R-15933, R-36351, LMG 32019^T^ and R-18634 had a significant inhibitory effect on mycelial growth (Figure 1b; *p* < 0.001). A differential response was observed between the strains as strain LMG 21824^T^ did not have an inhibitory effect under indirect contact (Figure 1b).

### 3.2 Phytotoxicity of the *Burkholderia* strains on tomato and wheat leaves

None of the strains displayed a phytotoxic effect on tomato leaf discs, evaluated as Fv/Fm and ChlIdx, after 24 h. On wheat leaves, LMG 21824^T^ and LMG 32019^T^ had a significant negative effect on Fv/Fm as compared to PBS control after 24 h (*p* = 0.012 and *p* = 0.008, respectively; Table 1) pointing to phytotoxicity or pathogenicity on wheat leaves.

**Table 1:** Chlorophyll fluorescence (Fv/Fm) and chlorophyll index (ChlIdx) of tomato (*Solanum lycopersicum* L. var. Marmande) leaf discs and wheat (*Triticum aestivum* L. var. Tybalt) leaflets inoculated with 5 μL of bacterial suspension (OD_600_ 2.0) and incubated for 24 h at 21 °C.

Strain	Cluster	Tomato		Wheat	
		Fv/Fm	ChlIdx	Fv/Fm	ChlIdx
PBS-control	–	0.716 ± 0.014	1.528 ± 0.126	0.706 ± 0.015	1.523 ± 0.059
LMG 21824^T^	Other Bcc A	0.707 ± 0.019	1.413 ± 0.182	0.663 ± 0.019*	1.418 ± 0.083
R-15933	Other Bcc A	0.719 ± 0.029	1.485 ± 0.174	0.709 ± 0.016	1.513 ± 0.092
LMG 32019^T^	Other Bcc F	0.714 ± 0.026	1.723 ± 0.397	0.671 ± 0.013**	1.490 ± 0.011
R-18634	Other Bcc F	0.707 ± 0.012	1.373 ± 0.228	0.694 ± 0.026	1.479 ± 0.107
R-36351	Other Bcc F	0.709 ± 0.019	1.426 ± 0.189	0.708 ± 0.027	1.442 ± 0.093

Values represent mean ± sd. of three biological replicates. Significant differences per proxy between the inoculated leaflets and PBS-control as per *t*-test are indicated with an asterisk (*p < 0.05; **p < 0.01).

### 3.3 *In vivo* biocontrol potential

**Figure 2:**
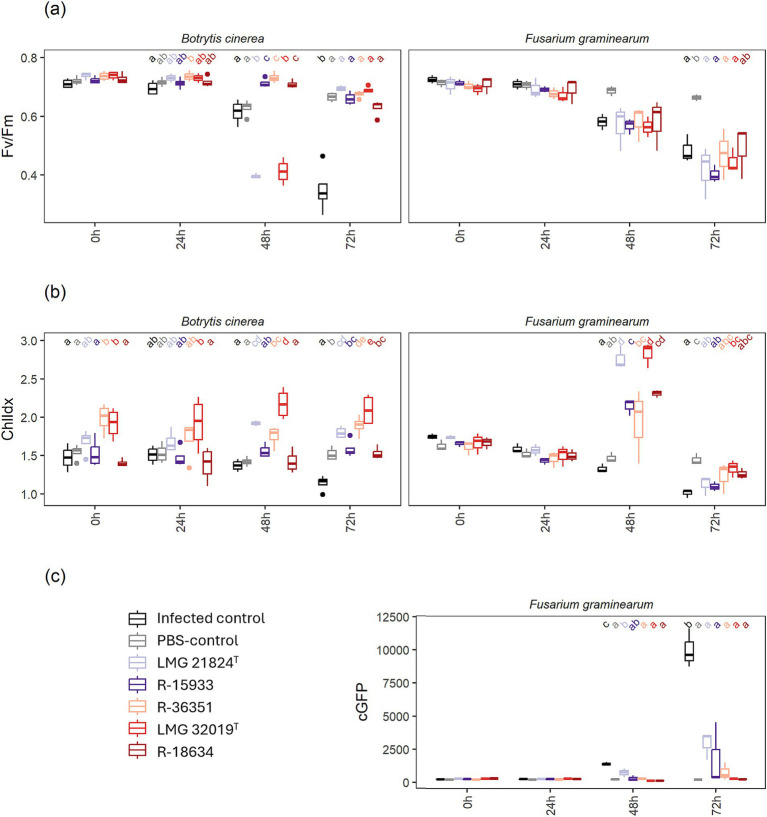
*In vivo* evaluation of plant health parameters upon co-inoculation of *B. cinerea* R16 on tomato leaf discs (left) and *F. graminearum* PH-1 on wheat detached leaves (right) with *Burkholderia* strains. **(a)** Chlorophyll fluorescence (Fv/Fm) as a proxy for photosynthetic efficiency (Fv/Fm-values range between 0 and 1, where values between 0.6 and 1 indicate healthy leaf tissue), **(b)** Chlorophyll index (ChlIdx) as a proxy for foliar chlorophyll content, and **(c)** cGFP-signal of *F. graminearum* PH-1 as a proxy for fungal biomass. Significant differences are indicated by means of significance letters per timepoint as per ANOVA and post-hoc Tukey test. Different letters indicate significant differences at a 95% confidence level.

**Figure 3:**
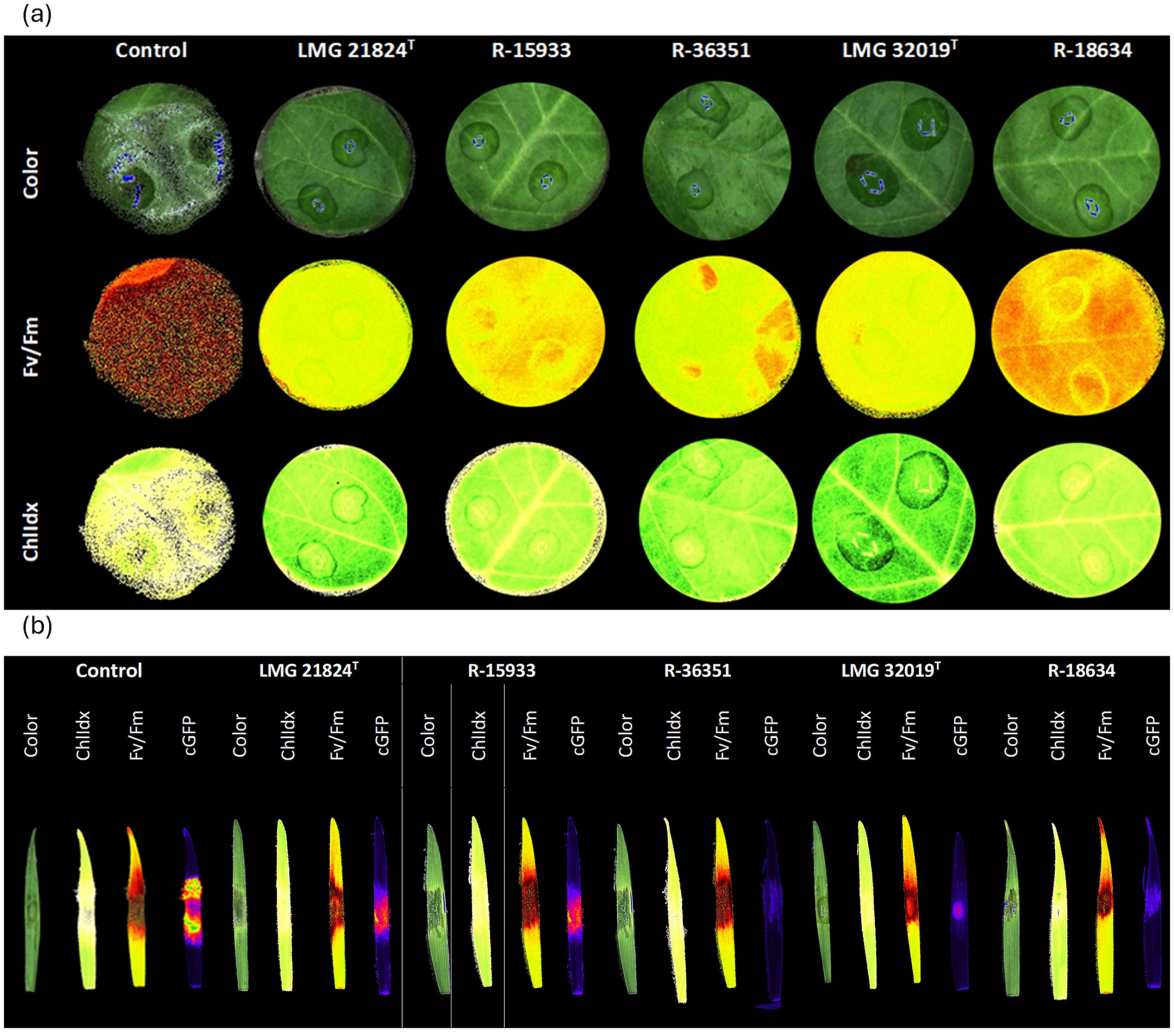
*In vivo* evaluation of plant health parameters upon co-inoculation of **(a)** *B. cinerea* R16 on tomato leaf discs with *Burkholderia* strains, and **(b)** of *F. graminearum* PH-1 on wheat leaves with *Burkholderia* strains. Color (high resolution images captured with multispectral imaging platform), chlorophyll fluorescence (Fv/Fm), and chlorophyll index (ChlIdx) images, and corrected GFP-fluorescence (cGFP) images.

In the tomato leaf disc assay, a significant decrease in chlorophyll fluorescence (Fv/Fm) values was observed throughout time in the *B. cinerea* R16-infected control discs (0 hpi, 0.712 ± 0.015; 72 hpi, 0.350 ± 0.083) (Figures 2a, 3a). Bacteria treated Bc-infected leaf discs had significantly higher Fv/Fm-values as compared to the infected control leaf discs at 72 h, pointing to a significant biocontrol effect, but no differential responses were observed between the different strains. Upon evaluation of the chlorophyll index (ChlIdx), significant differences between the uninfected and infected control leaf discs were observed at 72 h post infection (hpi). At this time point, all treatments had a significant inhibitory effect on the development of disease symptoms as measured by the ChlIdx compared to the infected control. Moreover, a significant differential response was observed for this proxy between the treatments, where strains LMG 21824^T^, LMG 32019^T^ and R-36351 had the highest ChlIdx values.

In the wheat detached leaf assay, a significant decrease in Fv/Fm values was observed at 72 hpi in the *F. graminearum* PH-1-infected control and in each of the infected leaves treated with *Burkholderia* strains as compared to uninfected control. There were no significant differences in Fv/Fm-values between the infected control leaves and the infected leaves treated with strains LMG 21824^T^, R-15933, LMG 32019^T^, R-36351 (Figure 2a). Upon evaluation of the ChlIdx, significant differences between the infected control leaves and infected leaves treated with strains LMG 21824^T^, R-15933, LMG 32019^T^, R-18634, and R-36351 were observed after 48 hpi. Except for strain LMG 32019^T^, this effect was transient after 72 hpi (Figure 2b). Upon evaluation of the cGFP-signal, there were significant differences between the infected control and all the treatments after 72 hpi. At this time point, all treatments significantly reduced the *F. graminearum* biomass accumulation as compared to the infected control (Figures 2c, 3b).

### 3.4 Whole genome sequencing, assembly, and annotation

The hybrid assembly of the ONT MinION long reads and the paired-end 150 bp Illumina reads resulted in assemblies of 3–5 contigs with N50 values ranging from 3,046 to 3,601 Mbp (Supplementary Table S3). The genomes ranged between 7.8–9.0 Mbp in size and were 99.94% complete and <1% contaminated. In addition to three principal replicons present in all strains, strains LMG 21824^T^ and R-36351 contained one and two plasmids, respectively. The raw ONT MinION long reads, the raw Illumina reads, and the annotated assemblies were submitted to the European Nucleotide Archive and are publicly available through the GenBank/EMBL/DDBJ under BioProject PRJEB76175 and the accession numbers listed in Supplementary Table S3.

### 3.5 Detection of biosynthetic gene clusters

**Figure 4:**
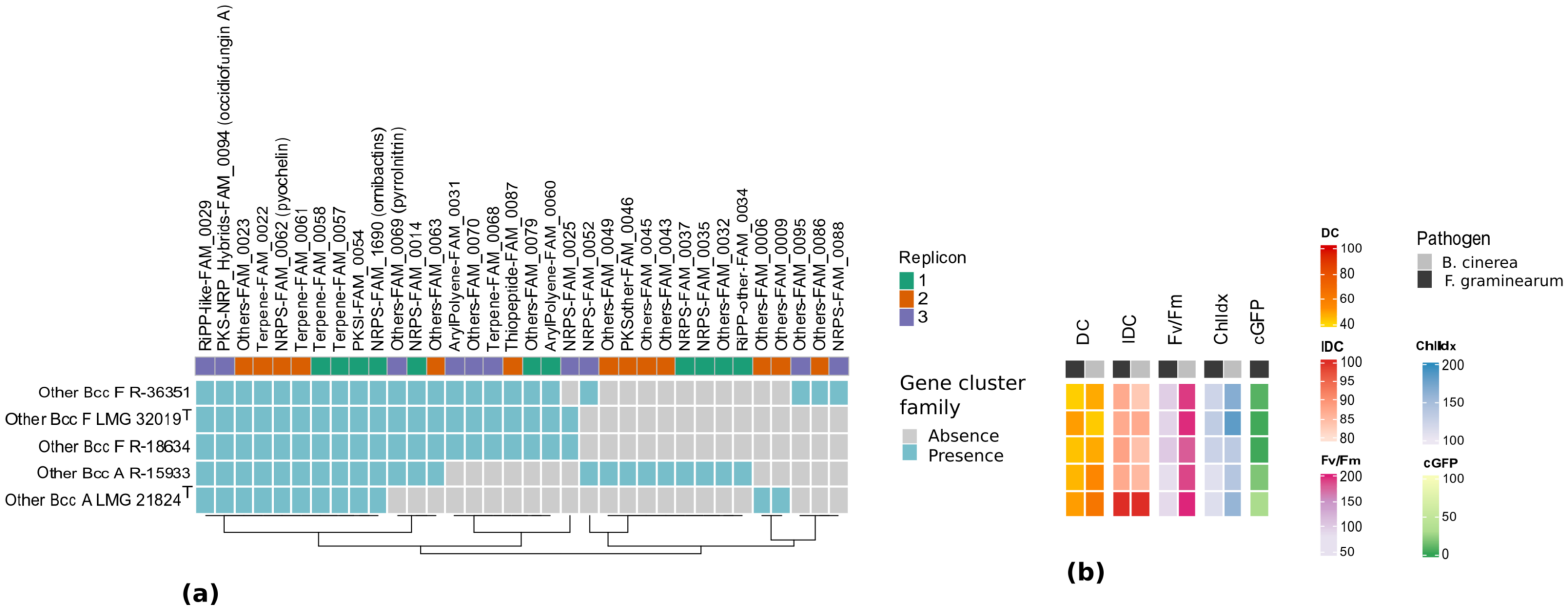
Correlation of secondary metabolite biosynthetic gene cluster families and antifungal activity of five *Burkholderia* strains. **(a)** Presence (cyan) or absence (gray) of gene cluster families (GCFs) in the *Burkholderia* genomes as determined via antiSMASH and BiG-SCAPE. Replicon positions of BGCs associated with each GCF are shown in green (replicon 1), orange (replicon 2) or purple (replicon 3) above the heatmap. Hierarchical clustering based on GCF presence/absence is represented by a dendrogram below the heatmap. **(b)** *In vitro* and *in vivo* biocontrol activity of *Burkholderia* strains against *B. cinerea* (gray) and *F. graminearum* (black). Values represent the percentage increase/decrease as compared to the untreated control [(mean_treatment_/mean_control_)*100]. DC, direct contact antagonism; IDC, indirect contact antagonism; Fv/Fm, chlorophyll fluorescence of wheat resp. tomato leaves; ChlIdx, chlorophyll index of wheat resp. tomato leaves; cGFP, corrected GFP signal correlated to the *F. graminearum* biomass. Increasing values correspond to a positive effect for DC, Fv/Fm, and ChlIdx, while decreasing values correspond to a positive effect for IDC and cGFP.

A total of 97 BGCs, all located on the three principal replicons, were detected across the five Bcc strains. Dereplication using BiG-SCAPE indicated that these 97 BGCs encoded 34 distinct gene cluster families (GCFs), each with a fully conserved replicon position (Figure 4). Four GCFs showed high similarity (>85%) with previously characterized gene clusters in the MIBiG database which encode antimicrobial compounds (Supplementary Table S4).

Ten GCFs were present in all five Bcc strains; these include the NRPS gene clusters for ornibactin (FAM_1690) on the first replicon, pyochelin (FAM_0062) on the second replicon, and the hybrid PKS-NRPS gene cluster for occidiofungin A (FAM_0094) on the third replicon (Figure 4). The remaining seven BGCs (four terpenes, one type 1 PKS, one RiPP-like and one phosphonate) had low or no similarity toward known gene clusters in the MIBiG database and were distributed across all three replicons (Supplementary Table S4).

Three GCFs (FAM_0069, FAM_0014 and FAM 0063) were present in all “Other Bcc F” strains and in “Other Bcc A” strain R-15933, but were absent in “Other Bcc A” LMG 21824^T^ (Figure 4). The BGCs in FAM_00069 were highly similar to the pyrrolnitrin gene cluster (MIBiG BGC0000924) and were located on the third replicon. FAM_0014 consisted of NRPS gene clusters located on the first replicon, where similarity toward chromobactin and aminochelin/azotochelin/protochelin gene clusters was 60–70% (MIBiG BGC0002679) and 50–62% (MIBiG BGC0002528), respectively (Supplementary Figure S2). The BGCs in FAM_0063 were identified as homoserine lactone gene clusters and were located on the second replicon (Supplementary Table S4).

Six GCFs were present in all three “Other Bcc F” strains and absent in both “Other Bcc A” strains (Figure 4). Two of those GCFs were located on the first replicon (isocyanide FAM_0079 and arylpolyene FAM_00060), one was located on the second replicon (thiopeptide FAM_0087), and three were located on the third replicon (arylpolyene FAM_0031, tRNA-dependent cyclodipeptide synthase [CDPS] FAM_0070 and a terpene FAM_0068).

Thirteen GCFs were strain-specific (Figure 4). The genome of “Other Bcc A” R-15933 contained eight unique GCFs, of which four were located on the first replicon (two NRPS, one homoserine lactone and one thioamitide) and four on the second replicon [two ectoines, one PKS-like and one opine-like metallophore BGC where similarity toward the pseudopaline gene cluster was 75% (MIBiG BGC0002489; Supplementary Table S4)]. The genome of “Other Bcc A” LMG 21824^T^ encoded two unique GCFs: FAM_0006 encoding a homoserine lactone BGC and FAM_0009, encoding a phenazine BGC. Finally, the genome of “Other Bcc F” R-36351 encoded three unique GCFs: FAM_0086 encoding a butyrolactone, FAM_0088 encoding an NRPS and FAM_0095 encoding a putative PKS-phenazine hybrid BGC with low similarity (48%) toward the esmeraldin gene cluster (MIBiG BGC0000935).

A clear species-level pattern was observed, in which Bcc F encoded six GCFs absent from all Bcc A strains, including thiopeptides, isocyanides, terpenes and two arylpolyene clusters (Figure 4). In contrast, Bcc A strains showed fewer species-specific GCFs, although R-15933 contained eight unique clusters absent in other strains.

### 3.6 Phylogenomic analysis

**Figure 5:**
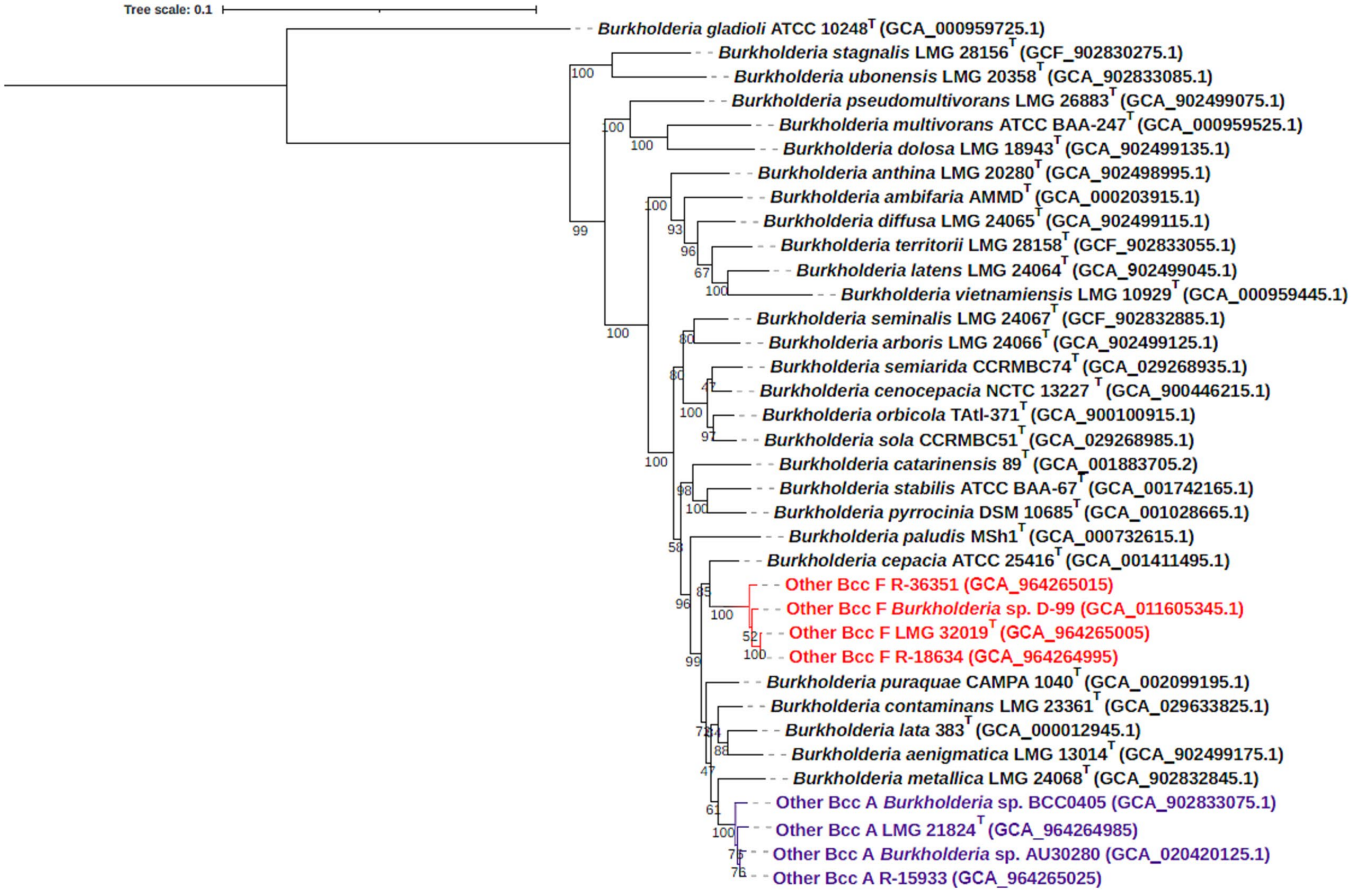
Maximum-likelihood bcgTree based on amino acid sequences of 107 core genes depicting the phylogenomic relationship between type strains of established BCC species, other BCC A and other BCC F strains, and *Burkholderia gladioli* ATCC 10248^T^. Bootstrap values are shown next to the branches. The scale bar indicates the number of amino acid substitutions per site. Novel species *Burkholderia crassaminum* sp. nov., (other BCC F, type strain = LMG 32019^T^), and *Burkholderia mycopellens* sp. nov. (other BCC A, type strain = LMG 21824^T^) are marked in red and purple font, respectively.

**Figure 6:**
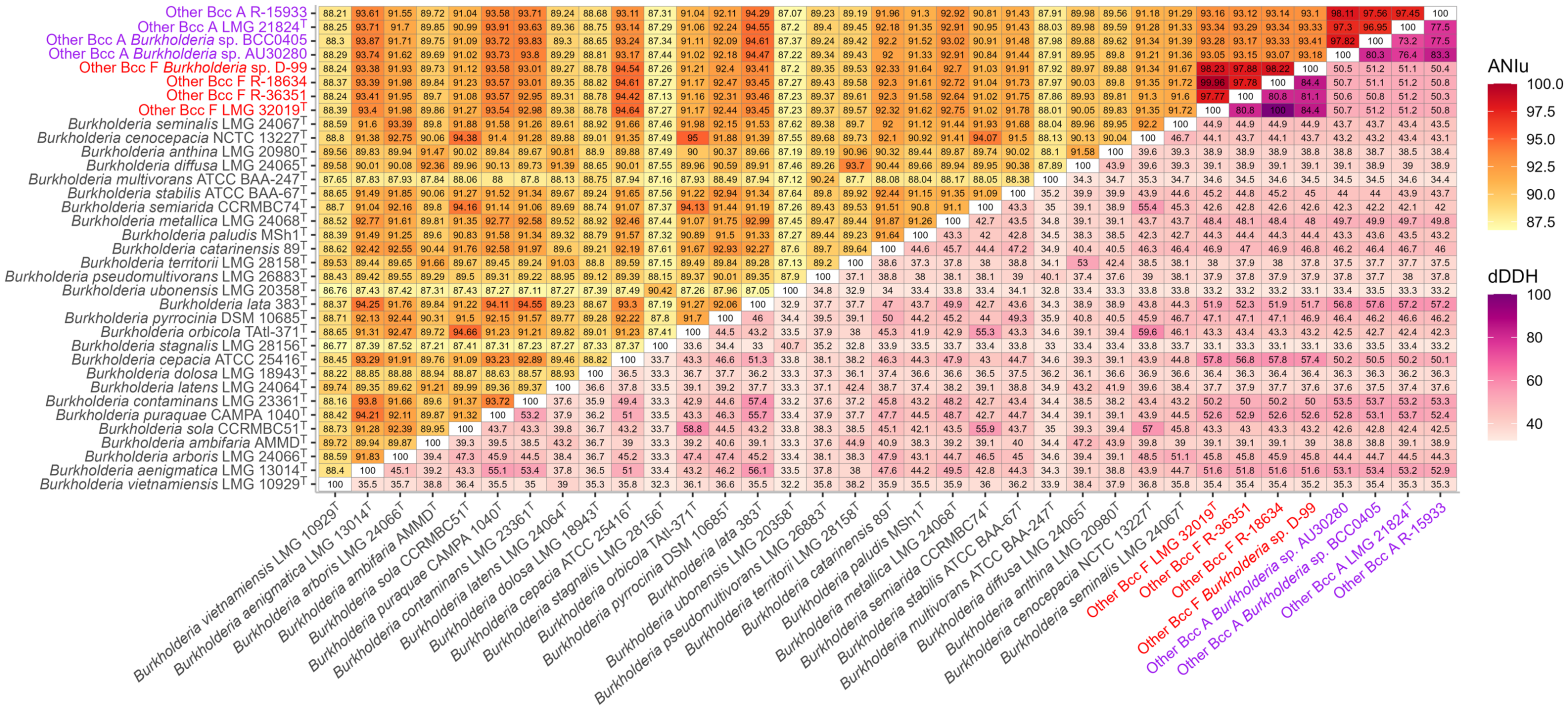
Pairwise ANI and dDDH values of other Bcc A, other Bcc F, and Bcc type strains.

GTDB-tk^3^ assigned strains LMG 21824^T^ to GTDB group “*Burkholderia* sp902833045” and LMG 32019^T^ to GTDB group “*Burkholderia* sp011605345.” A phylogenomic analysis based on 107 single-copy core genes confirmed that the five “Other Bcc A” and “Other Bcc F” strains were grouped into two distinct clades within the Bcc (Figure 5), each supported by a bootstrap value of 100. The two clades also encompassed the genome sequences of additional strains belonging to “Other Bcc group A” (*Burkholderia* sp. AU30280 and BCC0405) and F (*Burkholderia* sp. D-99) (Tatusova et al., 2014; Mullins et al., 2020). OrthoANIu and dDDH values between genomes within each clade were above the species delineation thresholds of 95–96 and 70%, respectively (Figure 6), and well below these species delineation thresholds toward the type strains of established Bcc species (Meier-Kolthoff et al., 2013a; Chun et al., 2018; Riesco and Trujillo, 2024).

### 3.7 Biochemical characterization and cellular fatty acid analysis

Biochemical characteristics and cellular fatty acid profiles were determined for two representative strains, i.e., “Other Bcc A” LMG 21824^T^ and “Other Bcc F” LMG 32019^T^, and are presented in Supplementary Table S5.

## 4 Discussion

*Burkholderia* strains commonly exhibit antifungal activity (Jung et al., 2018; Zeidan et al., 2019; Foxfire et al., 2021; Lin et al., 2021). Various modes of action by these strains have been reported, including direct competition, the production of antifungal compounds such as xylocandins, cepacidines, burkholdines, occidiofungins, and pyrrolnitrin, the production of volatile organic compounds, the disruption of the actin cytoskeleton, the induction of apoptosis in fungal cells, and the degradation of mycotoxins (Lu et al., 2009; Schmidt et al., 2009; Tawfik et al., 2010; Alvarez et al., 2022; Hansanant and Smith, 2022). In addition, *Burkholderia* strains support plant growth functions through multiple mechanisms, including the production of phytohormones, the stimulation of nutrient availability via nitrogen fixation and phosphate solubilization, and the production of 1-aminocyclopropane-1-carboxylate deaminase, which regulates ethylene levels and delays senescence (Liu et al., 2020; Heo et al., 2022; Pal et al., 2022).

In the present study, we analyzed the antifungal activity of *Burkholderia* strains LMG 32019^T^, R-18634, R-36351, R-15933, and LMG 21824^T^ against *B. cinerea* R16 and *F. graminearum* PH-1, both *in vitro* and *in vivo*. Earlier studies highlighted the usefulness of *Burkholderia pyrrocinia* CH-67, *Burkholderia vietnamiensis* B418, and *B. cepacia* Cs5 to control *B. cinerea* infections in tomatoes and vine (Kilani-Feki and Jaoua, 2011; Lee et al., 2011; Li et al., 2024). Similarly, *B. gladioli* strains KRS027 and KJ-34 exhibited broad-spectrum antifungal activity through direct antagonism and metabolite production that included volatile organic compounds, effectively suppressing *B. cinerea* (Wang D. et al., 2023; Yang et al., 2023). Additionally, several *Burkholderia* strains including *Burkholderia ambifaria* H8, *B. pyrrocinia* F12, and *B. cepacia* strains Bc-B and Bc-1 showed potential as biological control agents against *F. graminearum* in wheat and maize (Mao et al., 1998; Chen et al., 2024; Dan-Dan et al., 2024). In addition, *Burkholderia contaminans* KNU17BI1, KNU17BI3 and *Burkholderia cenocepacia* KNU17BI2 have been reported for their potent antifungal activity against several *Fusarium* species and their plant growth-promoting abilities in maize (Tagele et al., 2018; Tagele et al., 2019). In this study, mycelial growth of both fungi was strongly suppressed by all five *Burkholderia* strains when in direct contact (Figure 1a; Supplementary Figure S1). This inhibitory effect was also observed in the absence of direct contact for strains R-36351, LMG 32019^T^, R-18634, and R-15933, but not for strain LMG 21824ᵀ (Figure 1b; Supplementary Figure S1), suggesting the constitutive production of bioactive compounds in the former four strains.

The chlorophyll fluorescence (Fv/Fm) and chlorophyll index (ChlIdx) were used to assess the effect of the five strains on detached tomato and wheat leaves inoculated with *B. cinerea* and *F. graminearum, respectively.* The Fv/Fm was used as a proxy for the photosynthetic efficiency of the leaves and thus a measure for leaf health, while the ChlIdx was used as a proxy for foliar chlorophyll content and thus as a measure for leaf greenness (Gitelson et al., 2003; Baker, 2008). Both measurements revealed that each of the five *Burkholderia* strains significantly reduced *B. cinerea* symptom development on tomato leaflets (Figures 2, 3a). Specifically, after 24 h there was no significant decline in ChlIdx upon co-inoculation of *B. cinerea* with strain LMG 32019ᵀ as compared to the infected control leaves. After 48 h, strains LMG 32019ᵀ, LMG 21824ᵀ and R-36351 had an inhibitory effect on *B. cinerea* disease progression measured as ChlIdx, as compared to the infected control, indicating biocontrol effect against *B. cinerea* (Figure 2b). Chlorophyll-oriented effects have also been reported earlier for *B. ambifaria* XN08 in wheat plants infected with *Rhizoctonia cerealis* (An et al., 2022) and for *Burkholderia anthina* MYSP113 in sugarcane plants (Malviya et al., 2019).

In the *F. graminearum*-wheat bioassay, the Fv/Fm-values of the infected control leaves and leaves co-inoculated with the *Burkholderia* strains and *F. graminearum* were significantly lower as compared to the optimal control leaves. Using the Fv/Fm-values as a proxy for the development of disease symptoms, no biocontrol effects were observed (Figure 2a). However, upon evaluation of the cGFP-signal, which is a proxy for the GFP-tagged *F. graminearum* biomass, all strains were able to significantly reduce biomass accumulation in infected wheat leaves (Figures 2c, 3b), a phenomenon also reported for *B. pyrrocinia* F12 in wheat plants (Dan-dan et al., 2024). As the reduction in *F. graminearum* biomass did not translate in reduced disease symptoms in wheat leaflets, evaluated as Fv/Fm-values, we hypothesize that the *Burkholderia* strains might have triggered the biosynthesis of the myco- and phytotoxin deoxynivalenol (DON). A possible explanation for this observation lies in the known role of DON during infection. In the F. graminearum–wheat pathosystem, DON is the major necrotizing virulence factor responsible for chlorosis and reduced photosynthesis during the necrotrophic phase. Its biosynthesis is strongly induced by the accumulation of reactive oxygen species (ROS) (Audenaert et al., 2010; Audenaert et al., 2013; Tan et al., 2021; Wang D. et al., 2023). Hence, an increased production of DON in response to Burkholderia could explain why disease severity did not decrease, despite lower fungal biomass. Although this hypothesis requires targeted DON quantification for confirmation, it has been reported that antifungal Burkholderia strains induce ROS in planta (Wang D. et al., 2023), which could in turn stimulate DON biosynthesis by F. graminearum.

In summary, all strains were effective in the control *B. cinerea* R16 infections in tomato, as measured through both Fv/Fm (photosynthesis) and ChlIdx (chlorosis and necrosis). All strains were able to reduce the biomass accumulation of *F. graminearum* PH-1, but could not prevent *F. graminearum* infection symptom development. An evaluation of the phytotoxicity of the five *Burkholderia* strains revealed no effects on tomato, but for two strains, i.e., LMG 21824^T^ and LMG 32019^T^, a negative effect on chlorophyll fluorescence of wheat leaves was observed (Table 1), precluding these strains as safe biocontrol agents in this context. As the primary objective of this study was to identify *Burkholderia* strains with antifungal activities with the aim to further select specific functional metabolites produced by these strains (Alvarez et al., 2025; Elshafie and Camele, 2021; Bach et al., 2022), all strains provided a promising basis for future applications of their metabolites in agricultural production.

Biosafety considerations are of particular importance in the context of the *B. cepacia* complex, in which several opportunistic human pathogens are identified, and which is subjected to regulatory restrictions in many countries. Notably, one of the strains included in this study originated from a cystic fibrosis (CF) patient. Bcc strains from clinical origin, including those of CF-patients, require elevated biosafety containment and pose constraints for field applications, restricting their direct deployment as biocontrol agents. Hence, applying cell-free metabolites rather than live *Burkholderia*-bacteria could provide a safer and more realistic route toward practical biocontrol use.

To explore whether the observed phenotypic patterns could be linked to genetic differences in secondary metabolite capacity, we examined the BGCs encoded by these strains. AntiSMASH and BIG-SCAPE analyses detected a large number of BGCs (Figure 4; Supplementary Table S4), each with a fully conserved replicon position which may provide functional advantages (Kunakom and Eustáquio, 2019). Ten GCFs were conserved across all five strains suggesting they contribute to unknown baseline functional traits. These included a GCF that encodes the production of occidiofungin A, a well-known antifungal glycopeptide in *Burkholderia* strains with activity against plant pathogens (Wang et al., 2016; Jia et al., 2022), and GCFs that encode production of the siderophores ornibactin and pyochelin. Siderophores were previously shown to completely inhibit the spore germination in *B. cinerea, F. oxysporum*, *F. udum*, and *Aspergillus niger*, and degraded their mycelial hyphae by sequestering and limiting their access to iron (Sulochana et al., 2014; Esmaeel et al., 2016). Three additional GCFs including the pyrrolnitrin GCF were present in all strains except LMG 21824^T^. Pyrrolnitrin is a well-known antifungal compound with activity against a broad range of pathogens (el-Banna and Winkelmann, 1998; Sultan et al., 2008). Its primary target is the cell membrane, where it disrupts protein, RNA, and DNA synthesis, and interferes with the electron flow in the respiratory electron transport chain (Tripathi and Gottlieb, 1969; Pawar et al., 2019). While a role for the other strain LMG 21824^T^–specific GCFs (FAM_0063 and FAM_0014 GCFs) cannot be excluded, the lack of the pyrrolnitrin GCF may explain the weaker inhibitory activity observed in strain LMG 21824^T^.

A comparative analysis of BGC composition, organization, and genomic positioning across the five genomes revealed both conserved and lineage-specific biosynthetic architectures. Siderophore and antifungal-associated clusters, including ornibactin, pyochelin, and occidiofungin, were conserved and consistently localized to replicons 1, 2, and 3, respectively (Figure 4). In contrast, the remaining BGCs displayed lineage- and strain-specific patterns. “Other Bcc F” strains shared six additional GCFs absent from “Other Bcc A” strains, including isocyanide, aryl polyene, thiopeptide, CDPS, and terpene clusters, suggesting a lineage-specific expansion of secondary metabolite capacity. Conversely, the “Other Bcc A” strain R-15933 exhibited a distinct enrichment of unique NRPS-like, ectoine, and metallophore-associated clusters, highlighting substantial intra-lineage diversification. While most GCFs maintained conserved replicon positions across genomes, strain-specific clusters predominantly occurred on secondary replicons, supporting the hypothesis that accessory replicons act as evolutionary reservoirs for specialized metabolite diversification.

Six GCFs (Thiopeptide-FAM-0087, Others-FAM-0079, Others-FAM-0070, Terpene-FAM-0068, ArylPolyene-FAM-0031 and ArylPolyene-FAM-0060) were detected in “Other Bcc F” strains, but not in “Other Bcc A” strains (Figure 4; Supplementary Table S4) which may explain some weaker antifungal activities and less effective control of *F. graminearum* PH1 biomass compared to Bcc F strains (Figure 4b). In addition, 15 GCFs were variably present or strain specific. While this GCF diversity between strains pointed toward genetic adaptations which could influence their ecological roles and biotechnological potential (Kim et al., 2024), we did not observe a clear correlation with antifungal activity as determined in the present study.

The present study highlighted a diverse genetic background for the observed antifungal activities and provided a basis for further research into the mode of action of these *Burkholderia* strains. While GCFs encoding several well-known antifungal metabolites were detected, it did not provide a clear basis for explaining the phenotypic characteristics of these isolates. Given the observed phytotoxic effects on chlorophyll fluorescence of wheat leaves of strains LMG 21824^T^ and LMG 32019^T^, a further exploration of the mode of action of especially strains R-15933, R-36351, and R-18634 is necessary. The discovery of BGCs with low or no similarity to known clusters in the MIBiG database highlighted their potential as a source of novel bioactive compounds (Kunakom and Eustáquio, 2019).

Finally, we used the whole genome sequences to perform some key taxonomic analyses (Ankenbrand and Keller, 2016; Riesco and Trujillo, 2024). A phylogenomic analysis based on 107 single-copy core genes confirmed that “Other Bcc A” and “Other Bcc F” strains clustered into two distinct clades within the *B. cepacia* complex (Figure 5). The genomic relatedness of “Other Bcc A” and “Other Bcc F” strains toward type strains of established Bcc species was assessed by average nucleotide identity and pairwise digital DNA–DNA hybridization analyses (Figure 6). OrthoANIu and dDDH values among the four Other Bcc A strains ranged from 96.95 to 98.11% and 73.20 to 83.30%, respectively, while OrthoANIu and dDDH values between “Other Bcc A” strains and other Bcc strains were consistently below the species delineation thresholds of 95–96% ANI and 70% dDDH, respectively (Meier-Kolthoff et al., 2013b; Chun et al., 2018). Similarly, OrthoANIu and dDDH values among the four “Other Bcc F” strains ranged from 97.77 to 99.96% and 80.80 to 100%, respectively, while OrthoANIu and dDDH values between “Other Bcc F” strains and other Bcc strains were again consistently below the species delineation thresholds (Figure 6). These data unambiguously demonstrated that “Other Bcc A” and “Other Bcc F” strains each represent a novel Bcc species for which we propose the names *Burkholderia mycopellens* sp. nov., with LMG 21824^T^ (=CECT 30341^T^) as the type strain, and *Burkholderia crassaminum* sp. nov., with LMG 32019^T^ (=CECT 30343^T^) as the type strain.

## 5 Conclusion

In the present study, we demonstrated the *in vitro* and *in vivo* antifungal potential of five *Burkholderia* strains against *B. cinerea* and *F. graminearum*, highlighting their biocontrol effects on tomato and wheat plants under controlled conditions. Phytotoxicity assessments indicated limitations for two strains for biocontrol in wheat, which could be countered by using purified metabolites produced by the bacteria, instead of the living bacteria. Future work should compare the biocontrol potential of these *Burkholeria* strains with the biocontrol potential of (a selection of) their metabolites, thereby reducing the potential biosafety issues of working with *Burkholderia* isolates in agriculture. Although a validation in a greenhouse and field-setting is required, this study emphasizes the potential of *Burkholderia* strains for the selection of anti-fungal metabolites as biocontrol agents. Genome sequence analyses identified biosynthetic gene clusters linked to antifungal activity, but failed to provide a broad explanation of observed antifungal activities. Additionally, phylogenomic analyses supported the classification of these strains into two novel *B. cepacia* complex species for which we propose the names *Burkholderia mycopellens* sp. nov. and *Burkholderia crassaminum* sp. nov.

### 5.1 Description of *Burkholderia mycopellens* sp. nov.

*Burkholderia mycopellens* (my.co.pel’lens. Gr. n. mykês, a fungus; L. pres. part. *pellens*, chasing away, repelling; N. L. fem. Adj. mycopellens, chasing away fungi).

*Burkholderia mycopellens* cells are Gram-stain-negative, non-sporulating straight rods. Growth was observed on *B. cepacia* selective agar and MacConkey agar. Oxidase, lysine decarboxylase, esculin hydrolase, β-galactosidase and gelatinase activity are present. No ornithine decarboxylase, arginine dihydrolase, urease, and nitrate reductase activity is present. Growth is observed at 30 and 42 °C. Assimilation of L-arabinose, D-mannitol, N-acetyl-glucosamine, D-gluconate, glucose, caprate, adipate, citrate, and phenylacetate. No maltose assimilation is present. Acidification of maltose, lactose, D-xylose and adonitol; not of glucose and sucrose. The G + C content is between 65.83 and 66.5 mol%. The fatty acids identified were C_14:0_, C_12:0_, C_13:1_, C_16:0_, cyclo C_17:0_, C_16:0_ 2-OH, C_16:0_ 3-OH, C_16:1_ 2-OH, C_18:1_ 2-OH, C_18:1_ ω7c, and cyclo C_19:0_ ω8c. Summed feature 2 comprised C_14:0_ 3-OH, iso C_16:1_ I, an unidentified fatty acid with equivalent chain-length of 10.928 or C_12:0_ ALDE or any combinations of these components. Summed feature 3 comprised C_16:1_ ω7c or iso C_15_ 2-OH or both. *B. mycopellens* strains have been isolated from the environment and CF patients. The type strain is LMG 21824^T^ (= CECT 30341^T^). Phenotypic characteristics of the type strain are the same as described above for the species. Its G + C content is 66.09 mol% and its 16S rRNA and whole-genome sequences are publicly available through the accession numbers OZ204778 and GCA_964264985, respectively. Strain LMG 21824^T^ was isolated from cystic fibrosis patient in the United Kingdom.

### 5.2 Description of *Burkholderia crassaminum* sp. nov.

*Burkholderia crassaminum* (cras.sa.mi’num. L. gen. pl. n. *crassaminum,* from sediments).

*Burkholderia crassaminum* cells are Gram- stain-negative, non-sporulating straight rods. Growth was observed on *B. cepacia* selective agar and MacConkey agar. Oxidase, lysine decarboxylase, ornithine decarboxylase, β-galactosidase, nitrate reductase, and gelatinase activity are present. No arginine dihydrolase, urease, or aesculin hydrolase activity is present. Growth is observed at 30 and 42 °C. Assimilation of glucose, L-arabinose, D-mannose, D-mannitol, N-acetyl-glucosamine, D-maltose, D-gluconate, caprate, adipate, L-malate, citrate, and phenylacetate. Acidification of maltose, lactose, D-xylose and adonitol; not of glucose and sucrose. The G + C content is between 65.8 and 66.4 mol%. The following fatty acids are present: C_14:0_, C_12:0_, C_13:1_, C_16:0_, cyclo C_17:0_, C_16:0_ 2-OH, C_16:0_ 3-OH, C_16:1_ 2-OH, C_18:1_ 2-OH, C_18:1_ ω7c, cyclo C_19:0_ ω8c, summed feature 2 (comprising C_14:0_ 3-OH, iso C_16:1_ I, an unidentified fatty acid with equivalent chain-length of 10.928 or C_12:0_ ALDE or any combinations of these fatty acids), summed feature 3 (comprising C_16:1_ ω7c or iso C_15_ 2-OH or both). *B. crassaminum* strains have been isolated from the environment including river water, fresh water, freshwater sediments, and soil. The type strain is LMG 32019^T^ (= CECT 30343^T^). Phenotypic characteristics of the type strain are the same as described above for the species. Its G + C content is 66.4 mol% and its 16S rRNA and whole-genome sequences are publicly available through the accession numbers OZ204779 and GCA_964265005, respectively. Strain LMG 32019^T^ was isolated from river water in the United States.

## Data Availability

The datasets presented in this study can be found in online repositories. The names of the repository/repositories and accession number(s) can be found in the article/Supplementary material.

## Footnotes

1
https://github.com/nanoporetech/dorado

2
https://github.com/nanoporetech/medaka

3
https://github.com/Ecogenomics/GTDBTk
