# Supplementary material for: Biological control of Botrytis cinerea in tomato and Fusarium graminearum in wheat using the novel species Burkholderia mycopellens and Burkholderia crassaminum
Source: Front Microbiol. 2026 Apr 10;17:1776517. doi: 10.3389/fmicb.2026.1776517 (PMC13106412; doi:10.3389/fmicb.2026.1776517)
Supplement: Supplementary file 1 [file Table_1.docx]

Supplementary Material

# Supplementary Figures and Tables

## Supplementary Tables

**Table S1** Origin and isolation background of the Burkholderia-strains used in this research.

| **Strain** | **Isolation source** | **Depositor** | **Country** |
| --- | --- | --- | --- |
| LMG 21824^T^ | CF patient | John R. W. Govan | United Kingdom |
| R-15933 | Environment | John J. LiPuma | United States |
| LMG 32019^T^ | Freshwater | Eshwar Mahenthiralingam | United States |
| R-18634 | Soil | Eshwar Mahenthiralingam | United States |
| R-36351 | Freshwater sediment | John J. LiPuma | United States |

Table S2 Overview of the spectral indices used for the evaluation of plant health and fungal biomass.

| **Parameter** |  | **Formula** | **Physiological importance** | **Source** |
| --- | --- | --- | --- | --- |
| Chlorophyll fluorescence | $\frac{\text{F}_{\text{v}}}{\text{F}_{\text{m}}}$ | $\frac{\text{F}_{\text{m}}\text{- }\text{F}_{\text{0}}}{\text{F}_{\text{m}}}$ | Efficiency of photosystem II in a dark adapted state (PSII). Plant health measurement.  $F_{0}$: Minimal level of fluorescence measured after exposure to a weak measuring beam.  $F_{m}$: Maximum level of fluorescence measured after exposure to a brief saturating pulse. | (Baker 2008) |
| Chlorophyll index | ChlIdx | $\frac{\text{ρ}_{\text{770}}}{\text{ρ}_{\text{710}}}\text{- 1}$ | Leaf greenness. Vegetation index for the estimation of the chlorophyll content in leaves. | (Gitelson, Gritz et al. 2003) |
| GFP-fluorescence | cGFP |  | GFP fluorescence corrected for autofluorescence due to leaf senescence. |  |

**Table S3** Genomic characteristics of Other Bcc A and Other Bcc F strains. The sequencing data is available under the BioProject PRJEB76175. Genome size (bp), N50, and G + C content (%) were calculated using QUAST. The number of coding DNA sequences were determined using Prokka. Plasmids were identified using PlasmidHunter.

| **Strain** | **Genome**  **size (bp)** | **Replicon 1 (bp)** | **Replicon 2 (bp)** | **Replicon 3 (bp)** | **Plasmids (bp)** | **N50 (bp)** | **G + C content (%)** | **Coding DNA sequences** | **Assembly accession** |
| --- | --- | --- | --- | --- | --- | --- | --- | --- | --- |
| LMG 21824^T^ | 7,799,642 | 3,585,571 | 3,046,645 | 999,322 | 1 (168,104) | 3,046,645 | 66.09 | 7,009 | GCA_964264985 |
| R-15933 | 8,541,262 | 3,917,836 | 3,486,477 | 1,136,949 | No | 3,486,477 | 65.83 | 7,604 | GCA_964265025 |
| LMG 32019^T^ | 8,395,173 | 3,689,843 | 3,601,505 | 1,103,825 | No | 3,601,505 | 66.42 | 7,414 | GCA_964265005 |
| R-18634 | 8,395,221 | 3,689,806 | 3,601,574 | 1,103,841 | No | 3,601,574 | 66.42 | 7,414 | GCA_964264995 |
| R-36351 | 9,008,239 | 3,945,256 | 3,495,121 | 1,200,466 | 2 (170,182 and  197,214) | 3,495,121 | 65.80 | 8,155 | GCA_964265015 |

Table S4 Detection of biosynthetic gene clusters. Antismash_bigscape (separate dataset; excel-file)

Table S5 Biochemical data and cellular fatty acid profile of type strains Burkholderia mycopellens sp. nov., (Other Bcc A LMG 21824^T^) and Burkholderia crassaminum sp. nov. (Other Bcc F LMG 32019^T^). Only fatty acids that account for at least 1% of the total fatty acid profile of both strains are shown.

|  | **LMG 21824^T^** | **LMG 32019^T^** |
| --- | --- | --- |
| Growth on BCSA | + | + |
| Growth on MacConkey | + | + |
| Growth at 42°C | + | + |
| Pigment | y | - |
| Hemolysis (sheep blood) | - | + |
| Assimilation of L-arabinose | + | + |
| Assimilation of D-mannitol | + | + |
| Assimilation of N-acetyl glucosamine | + | + |
| Assimilation of maltose | - | + |
| Assimilation of adipate | + | + |
| Assimilation of caprate | + | + |
| Assimilation of citrate | + | + |
| Assimilation of L-malate | - | + |
| Assimilation of phenylacetate | + | + |
| Assimilation of glucose | + | + |
| Acidification of maltose | + | + |
| Acidification of lactose | + | + |
| Acidification of D-xylose | + | + |
| Acidification of sucrose/saccharose | - | - |
| Acidification of adonitol | + | + |
| Activity of lysine decarboxylase | + | + |
| Activity of ornithine decarboxylase | - | + |
| Nitrate reduction | - | + |
| Activity of aesculin hydrolysis | + | - |
| Activity of arginine dihydrolase | - | - |
| Activity of gelatinase | + | + |
| Activity of beta-galactosidase | + | + |
| Urease | - | - |
| Acidification of glucose | - | - |
| Indole production | - | - |
| **Fatty acid content** |  |  |
| C_14:0_ | 4.1 | *T |
| C_12:0_ | 2.2 | 6.27 |
| C_13:1_ | *T | *T |
| C_16:0_ | 16.7 | 18 |
| C_16:0_ 2-OH | 1.2 | 2 |
| C_16:0_ 3-OH | 5.9 | 5.8 |
| C_16:1_ 2-OH | 1.1 | 1.7 |
| C_17:0_ cyclo | 3.3 | 5.4 |
| C_18:1_ 2-OH | 2.4 | 2.6 |
| C_18:1_ ω7c | 33.7 | 30.3 |
| C_18:0_ | *T | 1.12 |
| C_19:0_ cyclo ω8c | 1.3 | 2 |
| **Summed feature 2 | 7 | 7.7 |
| ***Summed feature 3 | 20 | 16.2 |

*T trace amount (Trace amounts refer to fatty acids that each constitute less than 1% of the overall fatty acid profile).

**Summed feature 2 comprises C_14:0_ 3-OH, iso-C_16:1_ ISO I, an unidentified fatty acid with equivalent chain-length of 10,928 or C_12:0_ ALDE or any combinations of these.

***Summed feature 3 comprises C_16:1_ ω7c or C_15_ ISO 2-OH or both.

## Supplementary Figures


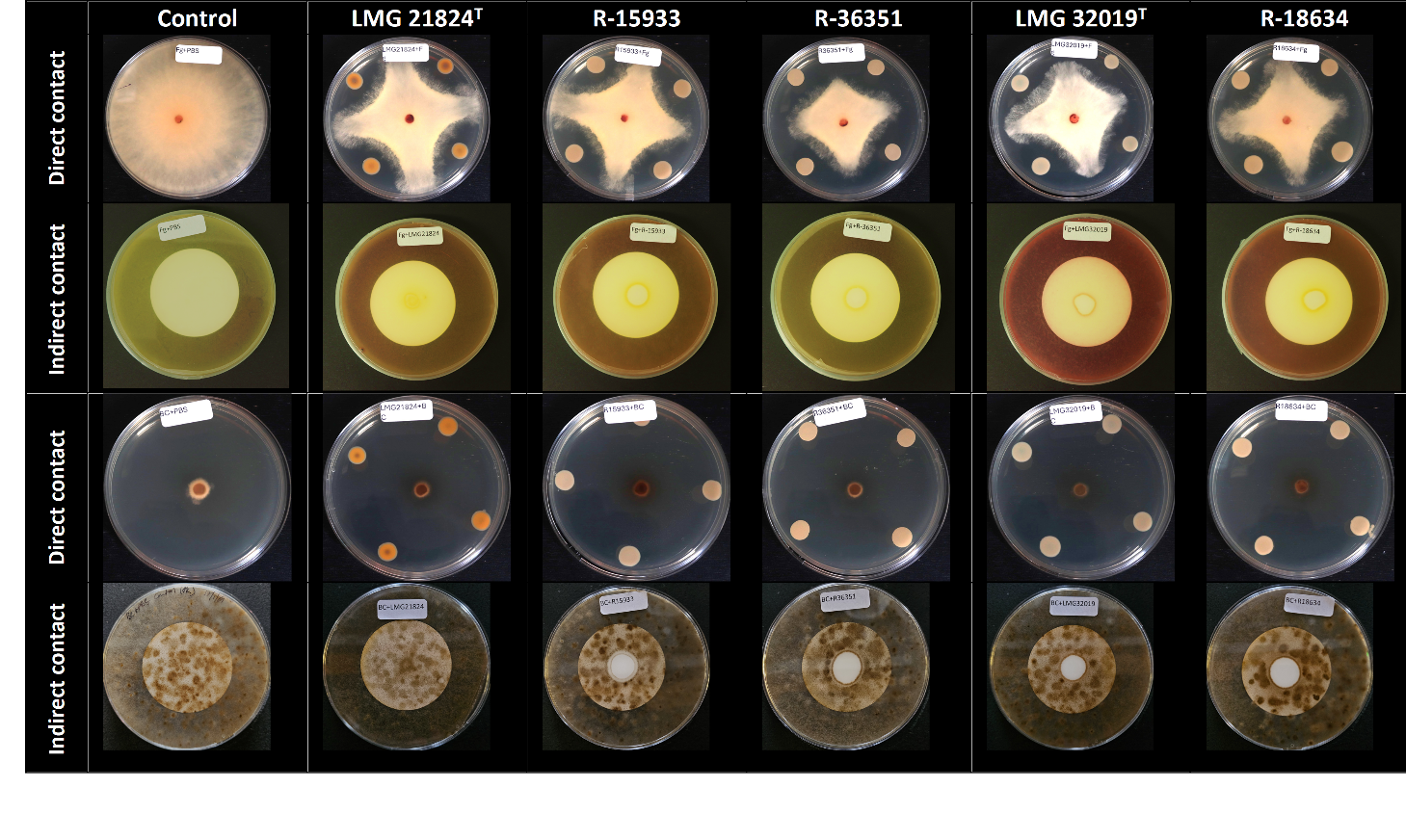


Figure S1 *In vitro* antagonism of *Burkholderia* isolates under direct and indirect contact against *F. graminearum* PH-1 (top), and under direct and indirect contact against *B. cinerea* R16 (bottom) at 48 hours post inoculation.


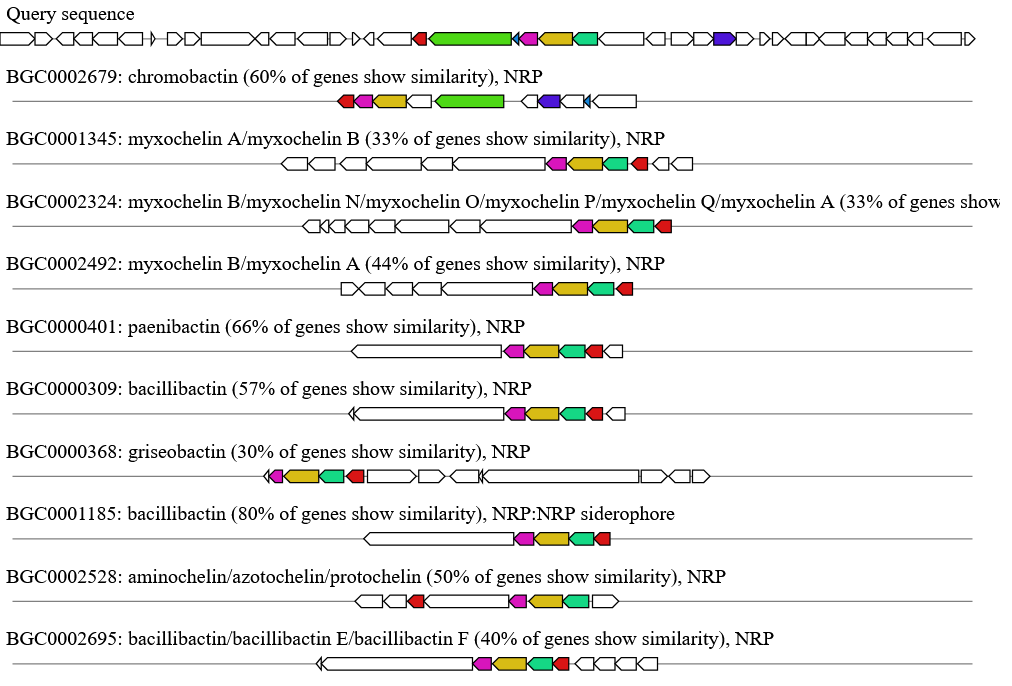


(a)


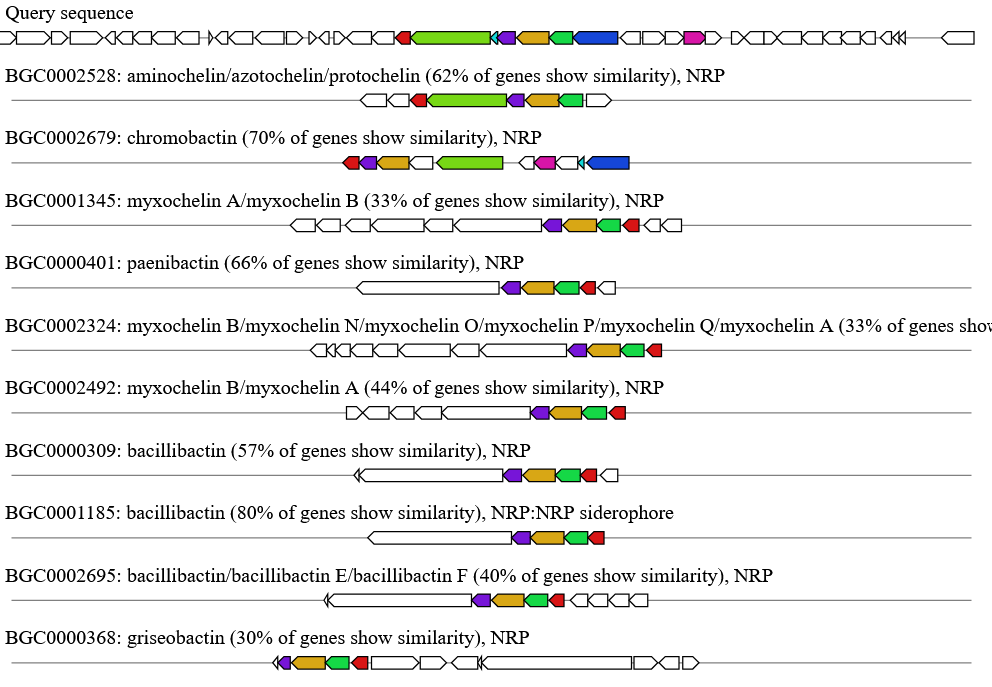


(b)


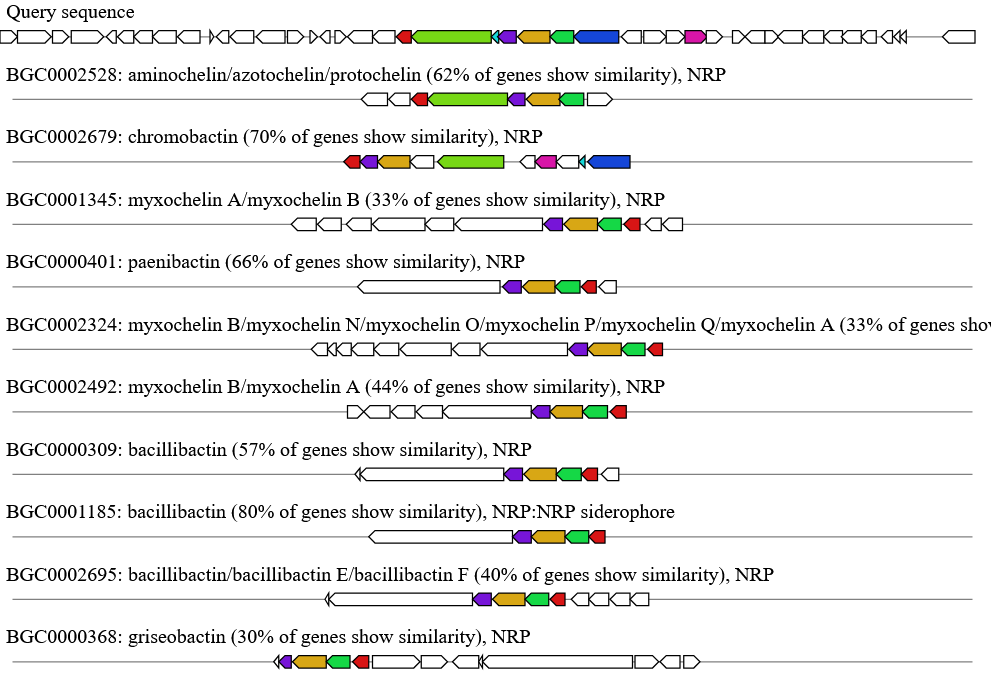


(c)


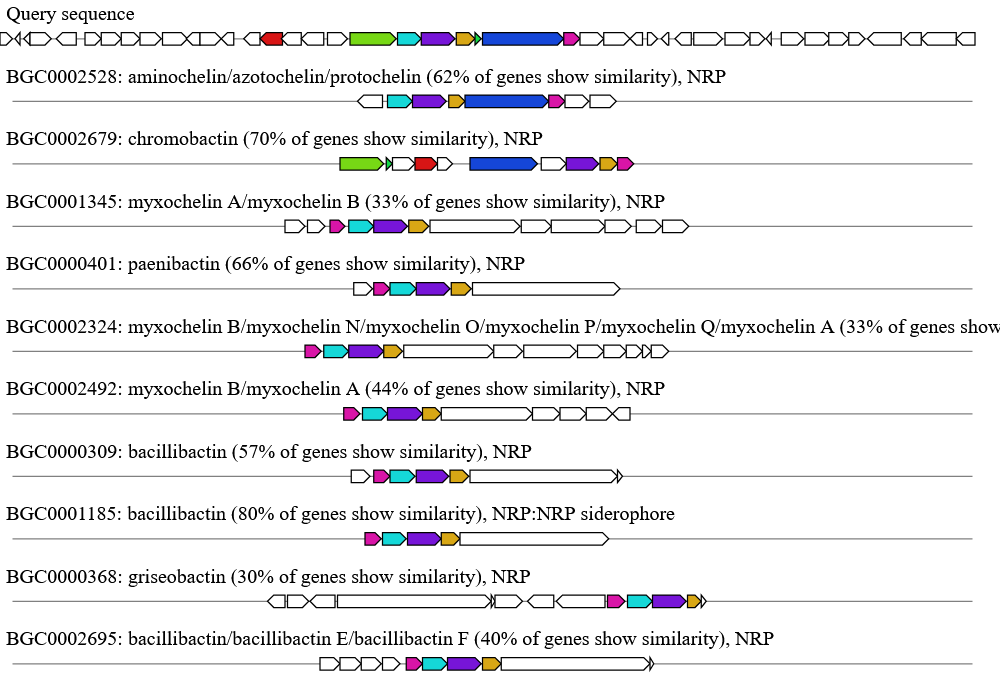


(d)

Figure S2 Overview of the knownClusterBlast of the FAM_0014 from strains (a) R-15933, KnownClusterBlast results for region 1.7 (replicon 1), (b) LMG 32019^T^, KnownClusterBlast results for region 1.3 (replicon 1), (c) R-18634, KnownClusterBlast results for region 1.3 (replicon 1), and (d) R-36351, KnownClusterBlast results for region 1.2 (replicon 1). KnownClusterBlast is a feature in antiSMASH (antibiotics and Secondary Metabolite Analysis Shell) used to compare predicted biosynthetic gene clusters (BGCs) with previously characterized clusters in the Minimum Information about a Biosynthetic Gene cluster (MIBIG) databases. Genes with matching colors are interrelated.

# References

Baker, N. R. (2008). "Chlorophyll fluorescence: a probe of photosynthesis in vivo." Annu. Rev. Plant Biol. **59**: 89-113.

Gitelson, A. A., Y. Gritz and M. N. Merzlyak (2003). "Relationships between leaf chlorophyll content and spectral reflectance and algorithms for non-destructive chlorophyll assessment in higher plant leaves." Journal of plant physiology **160**(3): 271-282.
